# Tricellulin facilitates colorectal cancer metastasis through activation of the TGFβ/SMAD2/3 signalling pathway

**DOI:** 10.3389/fonc.2025.1562976

**Published:** 2025-04-11

**Authors:** Wenfang Yang, Ruoxi Cheng, Mengbin Qin, Xiaoping Pan, Yanlin Tan, Kaoyan Feng, Jinxiu Zhang, Jiean Huang

**Affiliations:** Department of Gastroenterology, The Second Affiliated Hospital of Guangxi Medical University, Nanning, Guangxi, China

**Keywords:** colorectal cancer, tricellulin, TGFβ/Smad signaling, metastasis, epithelial-mesenchymal transition

## Abstract

**Background:**

Tricellulin belongs to the TAMP family of proteins and is primarily localized at the tricellular tight junctions. While its role in the progression of cancer has been reported, its importance in the progression of colorectal cancer (CRC) remains unclear.

**Objective:**

This study aimed to determine the function and mechanism of tricellulin in CRC progression.

**Methods:**

The proteins expression in cells and/or tissues was determined by Western blot, immunohistochemistry staining, and/or RT-qPCR analyses. The biological functions of tricellulin were investigated through *in vitro* assays (CCK-8, Transwell migration, and colony formation assays) and *in vivo* xenograft models. Tricellulin was significantly upregulated in CRC tissues compared to adjacent normal tissues. The expression of tricellulin was correlated with poor prognosis in patients with CRC.

**Results:**

*In vitro* assays showed that tricellulin enhanced CRC cell proliferation, migration, and invasion. Mechanistically, tricellulin activated the TGFb1/SMAD2/3 pathway, while TGFb1 reciprocally controlled the expression of tricellulin. Also, tricellulin promotes CRC cell migration/invasion through EMT. *In vivo* models confirmed that the overexpression of tricellulin facilitated tumor growth and activated the TGFb1/ SMAD2/3 pathway in CRC.

**Conclusion:**

Our findings demonstrate thatTricellulin promotes the metastasis of colorectal cancer by activating the TGF-β/SMAD2/3 signaling pathway, and TGF-β1 can reciprocally regulate the expression of tricellulin.We have revealed a novel mechanism by which tricellulin forms a positive feedback loop to promote the growth and metastasis of CRC. This mechanism provides novel insights into CRC progression and suggests potential therapeutic targets.

## Introduction

According to the International Cancer Research Agency, the global incidence of colorectal cancer (CRC) ranked third among all cancer types in 2022, with 895,000 related deaths worldwide (1). CRC is a multi-stage disease driven by accumulated genetic and epigenetic changes (2–4). Early-stage CRC, particularly before metastasis, has a favorable five-year survival rate of 90%. However, survival drops significantly to less than 10% in patients with late-stage CRC, indicating an unfavorable prognosis (5–8). Moreover, the 10-year survival rates decrease further as the disease progresses, and patients with stage IV have a 0% survival rate (8). Despite recent advances, the biological mechanisms underlying the initiation and progression of CRC remain unknown. A better understanding of the pathogenesis of CRC and its molecular mechanisms will help to more effectively prevent its progression.

Tight junctions are critical components of intercellular adhesion in epithelial and endothelial cells. They play essential roles in maintaining cell polarity (the “fence” function) and regulating paracellular communication (the “barrier” function) (9–12). Deterioration of tight junctions results in dysregulated cellular functions and disrupted cellular polarity, which is associated with altered proliferative capacity (13).

Tricellulin, also known as MARVELD2, is encoded by the TRIC gene (13). It is primarily localized at tricellular tight junctions, the contact site of three epithelial cells (14), and is one of the three members of the transmembrane and tetraspan membrane protein (TAMP) family (15). It is an integral membrane protein for tight junction formation (16).

Tricellulin is a tight junction network component, but its molecular function remains elusive. Accumulating evidence has linked altered expression and subcellular localization of tricellulin to the progression of a variety of malignancies. For instance, upregulated tricellulin is correlated with unfavorable clinical outcomes in patients with hepatocellular carcinoma, while downregulated tricellulin in intrahepatic cholangiocarcinoma was linked to a worse prognosis (17). In pancreatic cancer, nuclear translocation of tricellulin is associated with decreased tumor differentiation and poor prognosis (17, 18). Lymphatic metastasis has been shown to be significantly correlated with survival rate (19). Additionally, emerging evidence reveals that nuclear translocation of tricellulin is associated with poor tumor differentiation via the MAPK and PKC pathways (20). This observation is further supported by the finding that well-differentiated pancreatic cancers express high levels of tricellulin (21). Moreover, decreased tricellulin levels indicate a favorable prognosis (22). Our preliminary study revealed that tricellulin facilitates CRC cell migration and invasion *in vitro* (23). In our previous work with 98 CRC patients, we found that tricellulin was significantly upregulated in CRC tissues compared to normal tissues, and its expression was correlated with tumor distant metastasis, advanced TNM stage, and poor overall survival (23). Epithelial-mesenchymal transition (EMT) is a well-documented process that regulates cancer cell migration and invasion. However, whether tricellulin facilitates CRC progression by promoting EMT requires further investigation.

In this study, we have investigated the expression patterns and changes of tricellulin in CRC and examined the biological functions and molecular mechanisms of tricellulin in the pathogenesis of CRC.

## Materials and methods

### Patients and tissue samples

This study was approved by the Ethics Committee of the Second Affiliated Hospital of Guangxi Medical University (Approval No. 2024KV[0781]) and was conducted in accordance with the Helsinki Declaration. Written informed consent was obtained from all participants. The study cohort included 56 pairs of CRC and adjacent normal tissues, collected between September 2019 and September 2022 at the Second Affiliated Hospital of Guangxi Medical University. Among the 56 patients, there were 36 males (64.3%) and 20 females (35.7%), with ages ranging from 40 to 89 years old. There were 5 cases of stage I CRC, 18 cases of stage II CRC, 27 cases of stage III CRC, and 6 cases of stage IV CRC. All patients were naïve to treatment and had no known comorbidities. Tumor staging and histological diagnoses were conducted according to the National Comprehensive Cancer Network (NCCN) colorectal cancer classifications (24).

### Cell culture

HCT-116 cells were purchased from Shanghai Institute of Biochemistry and Cell Biology (Shanghai, China) and cultured in Dulbecco’s Modified Eagle Medium (DMEM, Meilun, China) supplemented with 100 units/ml penicillin, 100 μg/ml streptomycin, and 10% fetal bovine serum (FBS, Eva Cell, China) at 37°C in a humidified atmosphere with 5% CO_2_. Cells were authenticated and tested for mycoplasma contamination. A short hairpin RNA (shRNA) targeting tricellulin (5’-GATGAGCAGATTGCCACATCA-3’) was inserted into a pcDNA6.3-EGFP vector (Invitrogen). Lentivirus carrying control or tricellulin-targeting shRNAs were generated and titrated as previously described in HEK293T cells (23). HCT116 cells were transduced with resulting lentivirus to knock down tricellulin (Sh-tricellulin) or as a negative control (Sh-tricellulin-nc). In parallel, tricellulin was overexpressed in HCT116 cells using lentivirus carrying tricellulin-encoding gene (Lv-tricellulin). Cells transduced with control lentivirus were used as a negative control (Lv-tricellulin-nc).

To activate or inhibit the TGFβ1/SMAD pathway, cells were treated with an agonist (TGFβ1, 10 ng/ml) or antagonist (SB525334, 5 µM) (MedChemExpress, USA).

### Human CRC xenograft model

Male Balb/C nude mice aged 5-6 weeks (Guangxi Medical University Experimental Animal Center, Nanning, China) were randomly divided into 2 groups (n=5). Each mouse was subcutaneously injected with 5 × 10^6^ HCT116 cells (Lv-tricellulin-nc or Lv-tricellulin) in 200 μL of serum-free DMEM in the unilateral flank area. Tumor growth and body weight changes were monitored daily for 20 days. Tumor volume was calculated using the following formula: (L × W^2^)/2, where L and W represent tumor length and width, respectively. All mice were euthanized 20 days after injection, and tumors were collected for immunohistochemical (IHC) staining. Tumor weights were recorded after the tumors were collected. All animal experiments were approved by the Guangxi Medical University Institutional Animal Care and Use Committee.

### Western blot analysis

Cells were solubilized in radioimmunoprecipitation assay (RIPA) buffer supplemented with a protease and phosphatase inhibitor cocktail (Xinsaimei, China). Protein concentration was determined using the bicinchoninic acid method using a commercially available kit (Beyotime, China). Subsequently, 40 μg of total protein from each sample was separated by 10% sodium dodecyl sulfate-polyacrylamide gel electrophoresis and transferred to a polyvinylidene fluoride membrane (EMD Millipore, USA). Membranes were then incubated with skim milk at room temperature for 1 hour, followed by overnight incubation with primary antibodies at 4°C. Membranes were then incubated with goat anti-rabbit IgG (5151P, Cell Signaling, 1:20,000) or goat anti-mouse IgG (5257P, Cell Signaling, 1:20,000) at room temperature for 1 hour. Subsequently, membranes were developed using enhanced chemiluminescent and images were captured using an Odyssey infrared imaging system (LICORbio, USA). Relative protein expression levels were analyzed using ImageJ software (version 1.50i, National Institute of Health). Primary antibodies used in this investigation were as follows: anti-GAPDH (GB15004, Servicebio, 1:3,000), anti-tricellulin (48-8400, Thermo Fisher Scientific, USA, 1:1000), anti-TGFβ1 (R22797, Zenbio, 1:1,000), anti-phospho-SMAD2 (HA722443, Zenbio, 1:1,000), anti-phosph-SMAD3 (R380775, Zenbio, 1:1,000), anti-SMAD2/3 (Ab202445, Abcam, 1:1,000). Anti-E-cadherin (20874-1AP, Proteintech, 1:4,000), and anti-N-cadherin (GB121135, Servicebio, 1:100).

### Cell counting kit 8 assay

Cell proliferation was determined *in vitro* using a CCK8 assay (Dojindo, Japan) according to the manufacturer’s protocols, as previously described. In brief, cells were sub-cultured into 96-well plates at a density of 5,000 cells/well. Subsequently, cells were treated with the indicated stimuli for 72 hours, after which cell viability was determined by incubating cells with CCK8 solution at 37°C for 2 hours. Absorbance was measured at 450 nm using a microplate reader.

### Transwell invasion assay

Cell migration was examined using a Transwell system with polycarbonate filters (8 μm pore size) (Costar, USA). Briefly, cells (5x10^4^/well) along with treatments were loaded into the upper chamber of the Transwell. The lower chamber was filled with 600 μL of DMEM containing 20% fetal cattle serum. Cells were allowed to migrate for 48 hours at 37°C. Subsequently, the un-migrated cells in the upper chamber were removed with a cotton swab, and the migrated cells were fixed and stained with 0.1% crystalline violet. The stained cells were counted in five randomly selected fields per well at 200× magnification under an inverted microscope. ImageJ software was used to quantify the data.

### Cell clone formation assay

Cells (500/well) were sub-cultured into six-well plates and incubated with indicated molecule/compound for 24 hours. Medium was then replaced with normal growth medium, and the cells were allowed to grow for 2 weeks to form colonies. Then, the colonies were fixed and stained with 0.1% crystalline violet, and the number of colonies (containing more than 50 cells) was quantified using the ImageJ software.

### Cell scratch wound healing assay

A total of 1 × 10^6^ cells with representative tricellulin expression status in the logarithmic growth phase were seeded into a 6-well plate in triplicate and cultured to 100% confluence. A straight scratch was made perpendicular to the reference marking on the bottom of each well using a sterile 200 µL pipette tip. Cell debris were removed with three PBS washing, followed by the addition of culture medium containing 1% FBS. Cells were then treated with or without TGFβ1 (10ng/ml) or SB525334 (5 µM) for 24 hours. Images were captured at identical positions at the beginning (0 hour) and the completion (24 hours) of the experiment using an inverted microscope (CKX53, Olympus Corporation, Japan). Wound areas were determined using ImageJ software and the wound healing rates were calculated according to the formula: Wound healing rate = (wound area at 0 hour - wound area at 24 hours)/wound area at 0 hour × 100%.

### Immunohistochemical staining

Briefly, sections (4-5 µm) were deparaffinized in xylene and rehydrated in a series of ethanol solutions. Next, sections were incubated with primary antibodies against tricellulin (SAB1306444, Sigma, 1:100), TGFβ1 (R346599, Zenbio, 1:1,000), SMAD2/3 (RT1566; Huaan Biotech, 1:200), phospho-SMAD2 (CY5859, Abways, 1:200), phosph-SMAD3 (CY5859, Abways, 1:200), E-cadherin (20874-1AP, Proteintech, 1:200), and N-cadherin (GB121135, Servicebio, 1:500). After washing with PBS, sections were incubated with HRP-conjugated goat-anti rabbit IgG (PV-9000, ZSGB-BIO) for 30 minutes at room temperature. Immunoreactivity was visualized using a DAB substrate kit (ZLI-9018, ZSGB-BIO). Sections were examined and quantified under a microscope at 200× magnification by two researchers who were blinded to the study design. Immunostaining was quantified using the immunoreactivity score (IRS) system, which integrates the staining intensity (SI) and the percentage of positively-stained cells (PC) (25). The SI was graded on a 0-3 scale: 0 (negative staining), 1 (light brown), 2 (moderate brown), and 3 (dark brown). While the PC was categorized as: 0 (<5% positive cells), 1 (5-24% positive cells), 2 (25-49% positive cells), 3 (50%-74% positive cells), and 4 (≥75% positive cells). The final IRS was calculated by multiplying PC and SI sores and the resulting scores were used to categorize the expression of the proteins into high (IRS≥6) and low (IRS<6) groups.

### Real-time quantitative polymerase chain reaction analysis

Total RNA was isolated using an Eastep^®^ Super Total RNA Extraction Kit (LS1040, Promega) according to the manufacturer’s instructions. Complementary DNA was synthesized using a StarScript III All-in-one RT Mix with gDNA Remover (A230-10, Genstar), according to the manufacturer’s instructions. RT-qPCR analysis was conducted to determine the expression levels of target genes (*TRICELLULIN*, *TGFβ1*, *SMAD2*, and *SMAD3*) using Power SYBR Green Master Mix (A303-10, Genstar) on a StepOnePlus™ Real-Time PCR System (ThermoFisher Scientific). The relative level of target genes was normalized to the level of GAPDH mRNA using the 2^-ΔΔCt^ method. The representative primers used in this experiment are listed in **Table 1**.

**Table 1:** Primer sequences for RT-qPCR.

Gene	Sequence 5’ → 3’	
*TRICELLULIN*	Froward	ACAGATGATGAGCGAGAACGC
	Reverse	ATTCTCTCATGTTCCTGTCGGC
*TGFβ1*	Froward	CGCGTGCTAATGGTGGAAA
	Reverse	GCTGAGGTATCGCCAGGAAT
*SMAD2*	Froward	TATGGCGCTGGCCTGATCTT
	Reverse	AGTCATCCAGAGGCGGAAGT
*SMAD3*	Froward	AGTGACCACCAGATGAACCAC
	Reverse	CTCGCAGTAGGTAACTGGCT
*GAPDH*	Froward	AATCAAGTGGGGCGATGCTG
	Reverse	GCAAATGAGCCCCAGCCTTC

### Statistical analysis

Data were analyzed using GraphPad Prism software 8.0 (GraphPad Software, USA). Quantitative data were expressed as the mean ± standard deviation (SD). Categorical variables were analyzed using the Chi Square test. Spearman’s correlation analysis was used to evaluate the relationship between the expression levels of tricellulin and TGFβ1 or SMAD2/3 in CRC samples. Pairwise comparisons were made using student’s t-test and one-way analysis of variance was used for multiple comparisons. A *P* value <0.05 was considered statistically significant.

## Results

### Upregulation of tricellulin is associated with poor prognosis in CRC

**Figure 1:**
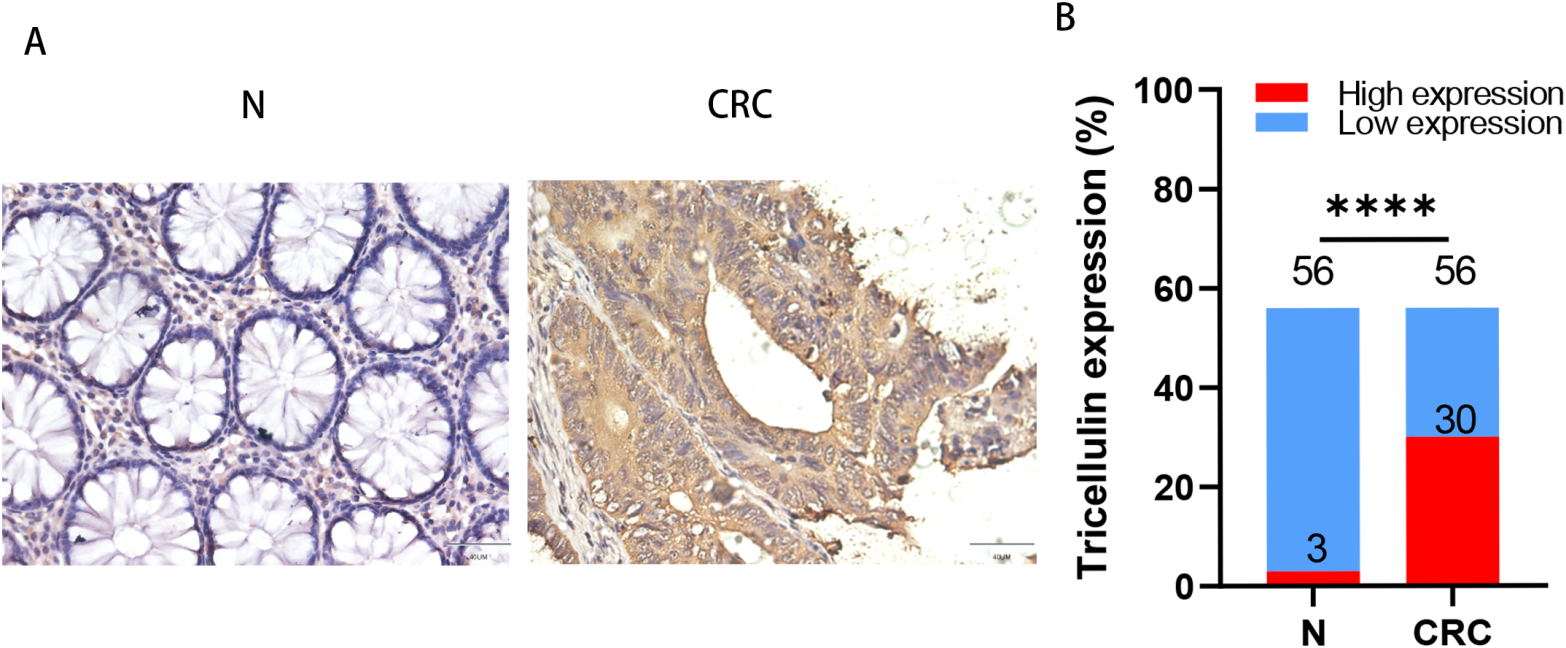
Tricellulin is upregulated in CRC tissues compared to normal colorectal tissues. Representative IHC images **(A)** and analyzed data **(B)** showing the expression levels of tricellulin in normal versus cancerous colorectal tissues. Scale bar = 20μm. n=56, ****P<0.0001.

To clarify the role of tricellulin in CRC progression, we analyzed its expression pattern and clinical significance in normal (n=56) and cancerous colorectal tissue (n=56) using IHC staining. The results demonstrated that the expression of tricellulin was significantly upregulated in CRC tissues compared to adjacent normal tissues (**Figure 1A**). Quantitative analysis of those tissues demonstrated that in normal colorectal tissues, only 3 individuals (5.36%) exhibited high levels of tricellulin. However, among CRC cases, 30 patients (53.6%) revealed high levels of tricellulin (**Figure 1B**).

To further determine the clinical importance of tricellulin, we investigated the relationship between tricellulin expression and the clinicopathological features. tricellulin expression was significantly correlated with tumor metastasis stage (M stages), tumor stages (TNM), and mismatch repair (MMR) status (proficient MMR (pMMR) and deficient MMR (dMMR)), but not with lymph node metastasis stages (N stages), gender, or age (**Table 2**). Together, these findings indicated that tricellulin may be involved in the progression of CRC.

**Table 2:** Correlation between tricellulin expression and clinicopathological characteristics of patients with CRC.

	Tricellulin expression		
Characteristics	High	Low	P-value
Gender			0.5508
Male	19	19	
Female	26	20	
Age			0.1331
≥ 65	27	17	
< 65	18	22	
N stage			0.1400
N0	17	21	
N1+N2	28	18	
M stage			0.0097
M0	15	24	
M1	30	15	
TNM stage			0.0027
I+II	12	23	
III+ IV	33	16	
MMR status			0.0052
pMMR	14	24	
dMMR	31	15	

### Tricellulin expression is correlated with TGFβ1 and SMAD2/3 in CRC

**Figure 2:**
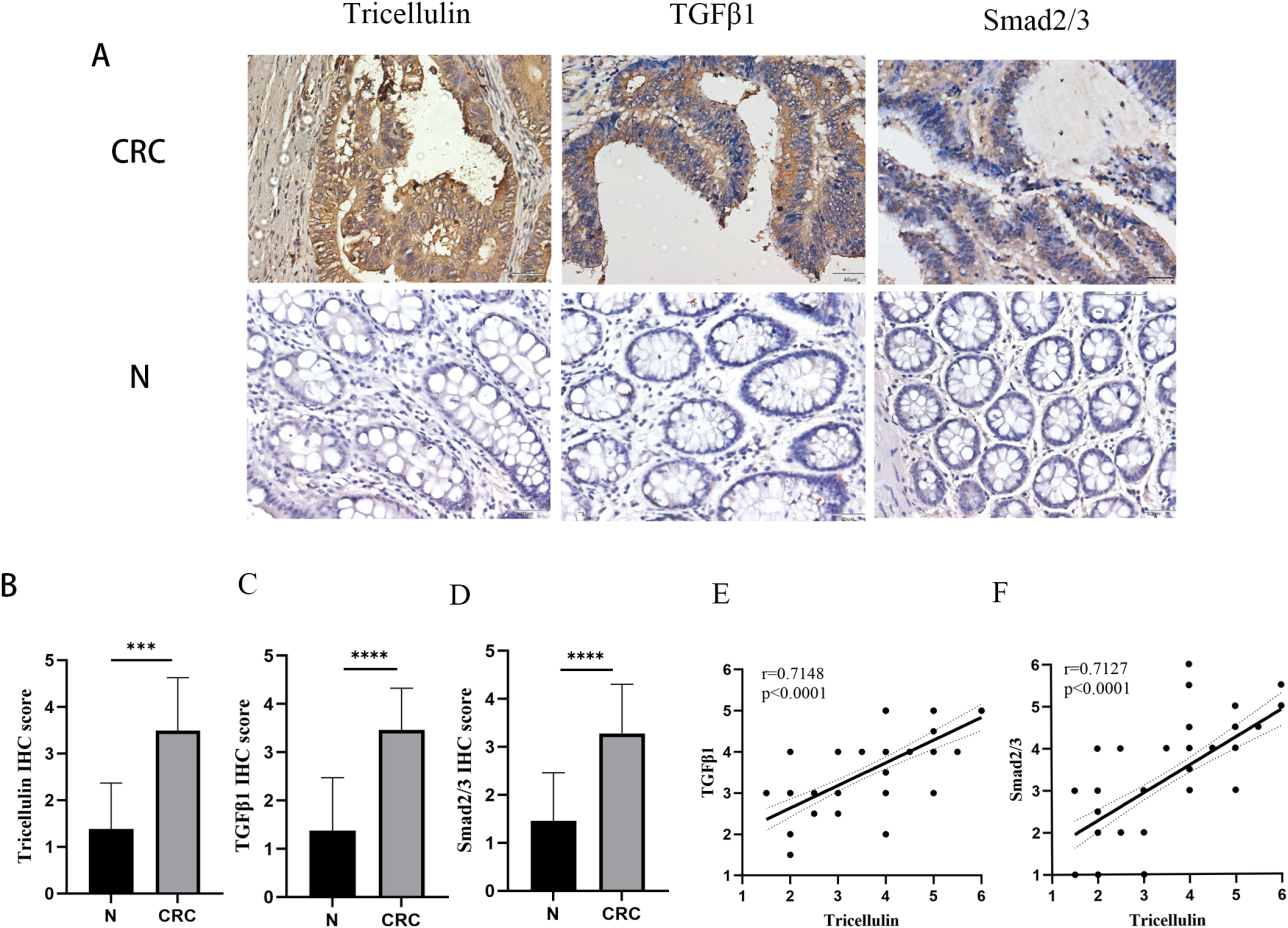
Tricellulin expressions correlated with TGFB1 and SMAD2/3 in CRC. Representative images of IHC staining demonstrating the expression of tricellulin, TGFβ1, and SMAD2/3 in normal and cancerous colorectal tissues **(A)**. Results of correlation analysis showing the positive correlation between tricellulin and TGFβ1 **(B)** and SMAD2/3 **(C)** in CRC tissues. Scale bar =20 μm. ***P<0.001, ****P<0.0001.

To explore the potential mechanisms by which tricellulin modulates CRC progression, we next examined the expression of TGFβ1 and SMAD2/3 in CRC and adjacent normal tissues using IHC staining. Both TGFβ1 and SMAD2/3 were significantly upregulated in CRC tissues compared to normal tissues (**Figure 2A**). Meanwhile, to further interrogate the potential regulatory role of tricellulin in CRC, we performed a correlation analysis. The results revealed that tricellulin expression was positively correlated with TGFβ1 (r=0.6334, *P*<0.0001) and SMAD2/3 (r=0.7396, *P*<0.0001) in cancerous tissues (**Figures 2B, C**). These findings suggest that the TGFβ1/SMAD2/3 pathway is involved in tricellulin-mediated CRC progression.

### Tricellulin promotes CRC cell proliferation, migration, and EMT *in vitro*

**Figure 3:**
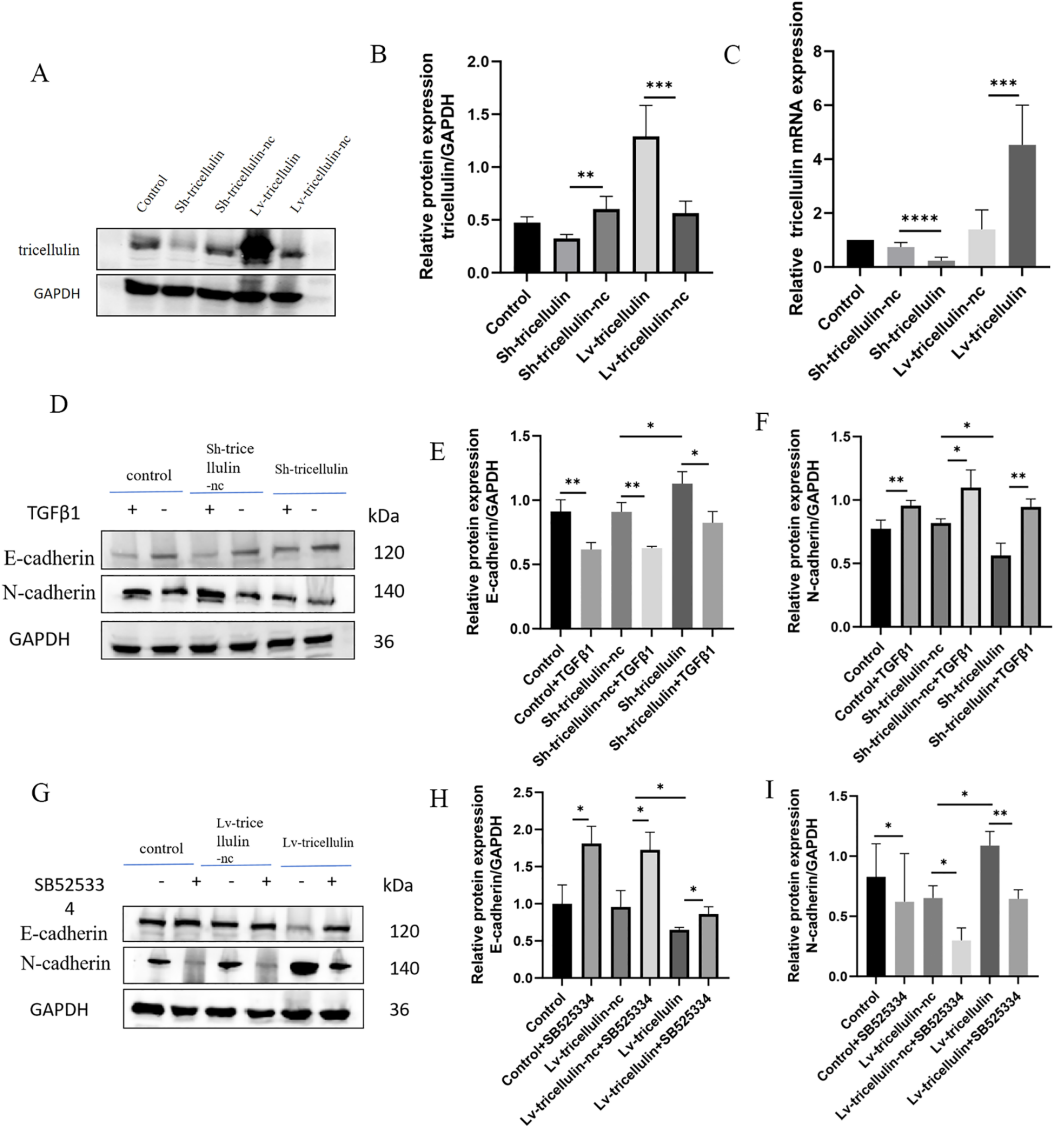
The effect of tricellulin on epithelial-mesenchymal transition in colorectal cancer cells. Representative images and quantified results of Western blot and RT-qPCR analyses confirming the overexpression and knockdown of tricellulin. **(A-C)** Representative images of Western blot analysis showing expression levels of EMT markers in HCT116 cells under indicated conditions **(D-I)**. n=3, *P<0.05, **P<0.01, ***P<0.001, ****P<0.0001.

Based on the clinical correlation between tricellulin and TGFβ1/SMAD2/3, we subsequently performed functional analyses to explore the biological functions of tricellulin in CRC *in vitro*. HCT116 cells with stable tricellulin knockdown (Sh-tricellulin) or overexpression (Lv-tricellulin) were established and confirmed by RT-qPCR and Western blot analyses, respectively (**Figures 3A–C**).

To examine whether tricellulin influences EMT in CRC, we determined the expression of EMT markers by Western blot analysis. Compared to the positive control (control) and negative control (Sh-tricellulin-nc) groups, tricellulin knockdown downregulated the mesenchymal marker N-cadherin while upregulating the epithelial marker E-cadherin. This EMT-suppressive effect required TGFβ1 (**Figures 3D–F**). Notably, this EMT-suppressive effect was partially reversed by the treatment of TGFβ1. Conversely, tricellulin overexpression elevated N-cadherin and decreased E-cadherin expression. This EMT-promoting effect was abolished by treatment with SB525334, a TGFβ1 signaling inhibitor (**Figures 3G–I**). Collectively, these observations suggest that tricellulin regulates EMT through the TGFβ1 signaling pathway.

**Figure 4:**
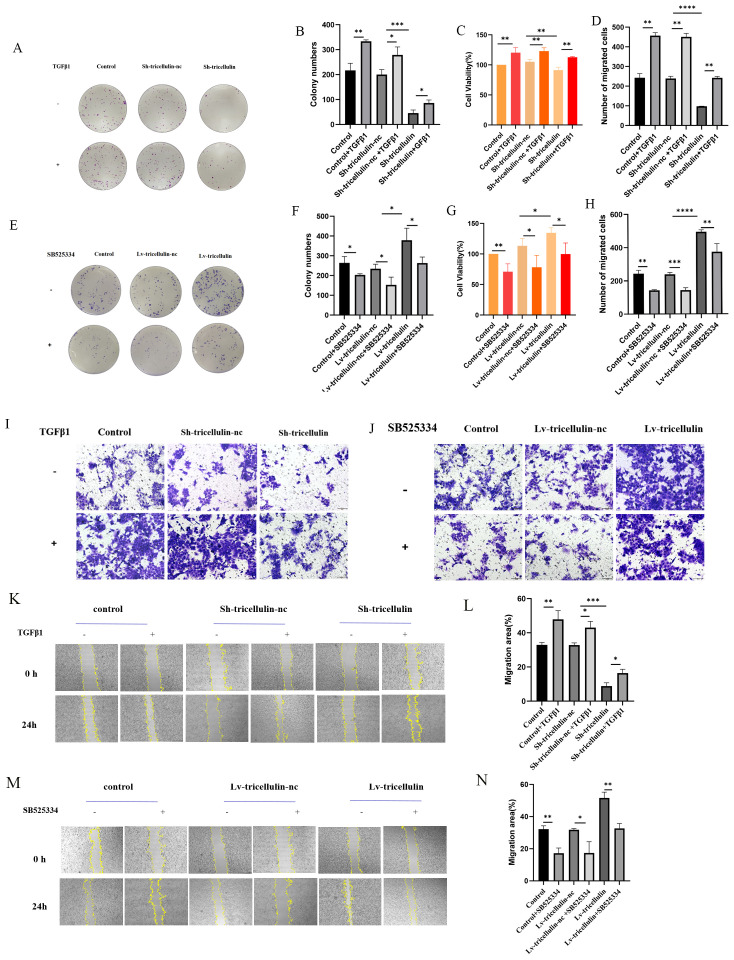
Tricellulin promotes cell proliferation and migration in CRC *in vitro*. Representative images and quantification of colony formation assays in HCT116 cells after tricellulin knockdown **(A, B)** and overexpression **(E, F)** under the indicated treatments. **(C, G)**. Quantified results of a CCK8 assay in HCT116 cells after tricellulin knockdown **(C)** or overexpression **(G)** under the indicated treatments. Representative images and analyzed data showing HCT116 cells after tricellulin knockdown **(D, I)** or overexpression **(H, J)** under the indicated treatments. Representative images and quantification of wound-healing assay in HCT116 cells after tricellulin knockdown **(K, L)** and overexpression **(M, N)** under the indicated treatments. **(C, G)**. n=3, *P<0.05, **P<0.01, ***P<0.001, ****P<0.0001.

To further determine the functional importance of tricellulin on CRC cell proliferation, migration, we conducted colony formation, Transwell migration, and wound healing assays. We found that tricellulin knockdown significantly suppressed colony-forming ability, cell viability, and cell migration compared to positive control and Sh-tricellulin-nc control groups (**Figures 4A–D, I, K, L**). However, TGFβ1 treatment reduced these tricellulin knockdown-mediated suppressive effects (**Figures 4A–D, I**). On the contrary, tricellulin overexpression significantly facilitated cell growth, viability, and migration in HCT116 cells (**Figures 4E, H, J, M, N**), while SB525334 significantly attenuated these pro-tumorigenic effects. Together, these observations indicate that tricellulin plays an essential role in CRC cell functions and that its regulatory roles are mediated through TGFβ1/SMAD2/3 signaling.

### Tricellulin regulates the TGFβ/SMAD2/3 signaling pathway in CRC cells

**Figure 5:**
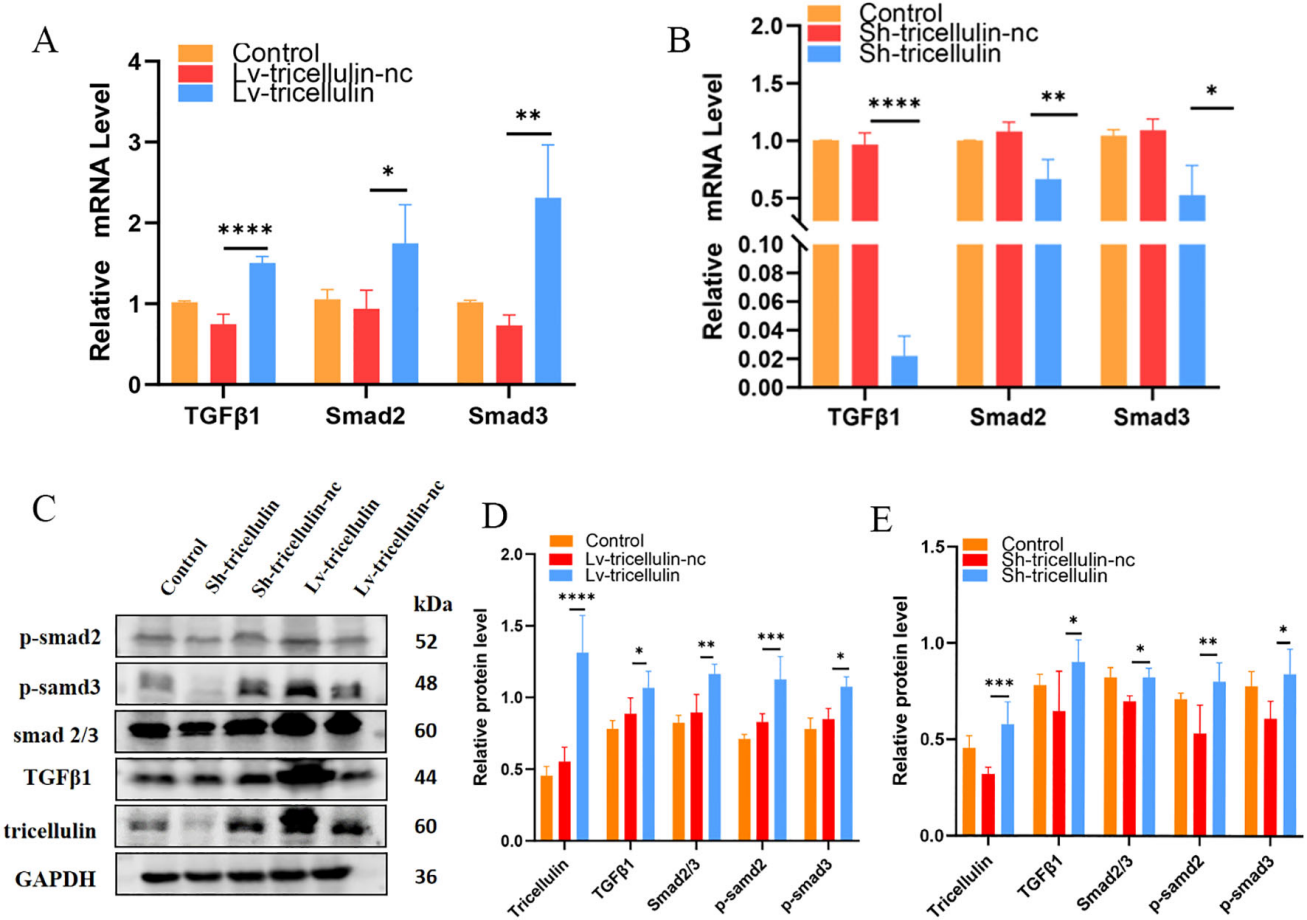
Tricellulin regulates TGFβ/SMAD2/3 signaling pathway in CRC cells. Results of RT-qPCR showing the relative expression levels of TGFβ1, SMAD2, and SMAD3 under the indicated conditions **(A, B)**. Representative images **(C)** and quantified results **(D, E)** of Western blot analysis demonstrating the expression and/or activation of TGFβ1, SMAD2/3, p-SMAD2, and p-SMAD3 under the indicated conditions. n=3, *P<0.05, **P<0.01, ***P<0.001, ****P<0.0001.

To further confirm the regulatory relationship between tricellulin and the TGFβ1/SMAD2/3 signaling pathway, we measured the expression and/or activation of each component at both mRNA and protein levels. At the mRNA level, TGFβ1 and SMAD2/3 transcription were significantly upregulated following tricellulin overexpression, and significantly downregulated following tricellulin knockdown (**Figures 5A, B**). Western blot analysis confirmed these findings, demonstrating elevated TGFβ1, SMAD2/3, and phosphorylation of SMAD2/3 upon tricellulin overexpression while tricellulin knockdown decreased their expression (**Figures 5C, D**). Together, these observations suggested that tricellulin functions as an upstream modulator of TGFβ1/SMAD2/3 pathway regulating CRC cell functions.

**Figure 6:**
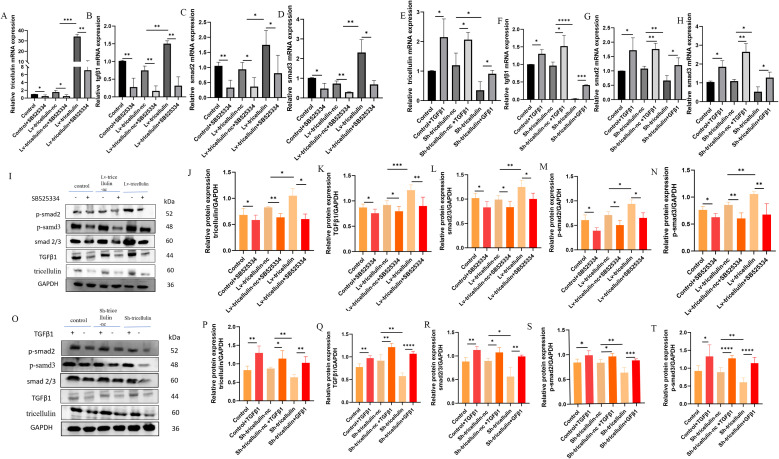
Tricellulin modulates TGFβ/SMAD2/3 signaling in CRC cells. Results of RT-qPCR demonstrate the expression of tricellulin **(A)**, TGFβ1 **(B)**, SMAD2 **(C)**, and SMAD3 **(D)** in CRC cells with or without tricellulin overexpression and treated with SB525334 or vehicle. Results of RT-qPCR showing the expression of tricellulin **(E)**, TGFβ1 **(F)**, SMAD2 **(G)**, and SMAD3 **(H)** in CRC cells with or without tricellulin knockdown and treated with TGFβ1 or vehicle. Representative images **(I)** and quantified results of Western blot analysis demonstrating protein levels of tricellulin **(J)**, ββ1 **(K),** total SMAD2/3 **(L)**, phosphorylated SMAD2 **(M)**, and phosphorylated SMAD3 **(N)** in CRC cells with or without tricellulin overexpression and treated with SB525334 or vehicle. Representative images **(O)** and quantified results of Western blot analysis demonstrating protein levels of tricellulin **(P)**, TGFβ1 **(Q)**, total SMAD2/3 **(R)**, phosphorylated SMAD2 **(S)**, and phosphorylated SMAD3 **(T)** in CRC cells with or without tricellulin depletion treated with TGFβ1 or vehicle. n=3, *P<0.05, **P<0.01, ***P<0.001, ****P<0.0001..

To further dissect the regulatory relationship between tricellulin and TGFβ1/SMAD2/3 pathway, we examined whether TGFβ1 signaling reciprocally regulates tricellulin. We observed that expression of tricellulin, TGFβ1, SMAD2, and SMAD3 in CRC cells were significantly reduced by SB525334 and elevated by TGFβ1 (**Figures 6A–H**). Notably, we also observed that inhibiting TGFβ signaling reduced tricellulin expression in CRC cells, while activating TGFβ signaling promoted tricellulin expression (**Figures 6A, E, I, J, O, P**). These observations suggest a positive feedback loop between tricellulin and the TGFβ1/SMAD2/3 signaling pathway in CRC cells.

### Tricellulin promotes CRC growth *in vivo*

**Figure 7:**
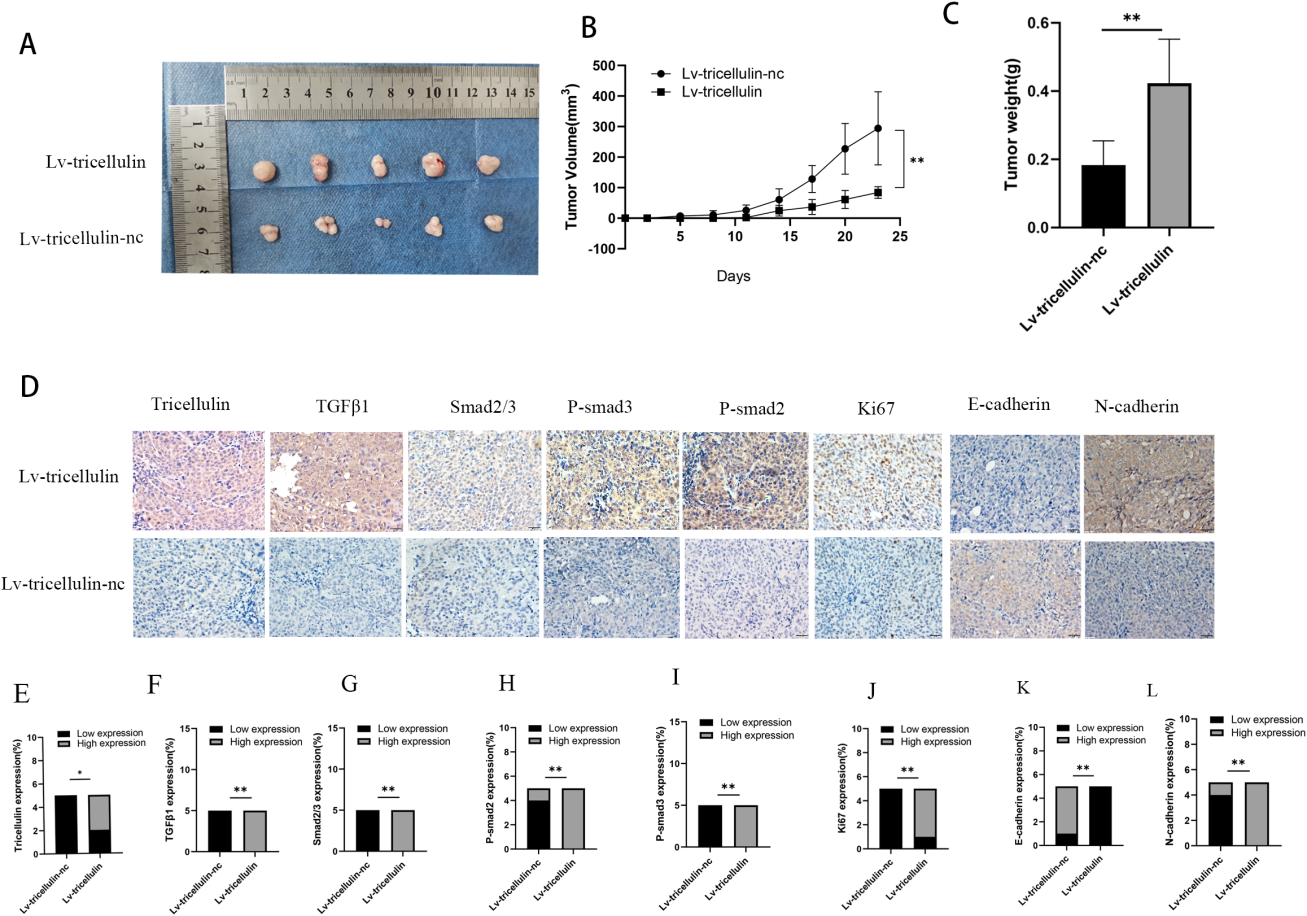
Tricellulin promotes CRC growth *in vivo*. Images of xenograft tumors from mice inoculated with HCT116 cells under indicated modulations **(A-C)**. Quantification of final tumor weights from indicated groups **(D)**. Tumor growth curves showing tumor volumes after cell implantation. Representative images of IHC staining of tricellulin, TGFβ1, SMAD2/3, and Ki67 in xenograft tumors **(F)**. Quantified expression levels of IHC staining intensity for tricellulin **(G)**, TGFβ1 **(H)**, SMAD2/3 **(I)**, and Ki67 **(J)**. Scale bar = 20 μm. n=5, *P<0.05, **P<0.01.

To evaluate the importance of tricellulin in the CRC pathogenesis *in vivo*, a xenograft model was established by subcutaneously inoculating HCT116 cells overexpressing tricellulin (Lv-tricellulin) or control cells (Lv-tricellulin-nc). Tumor growth was significantly facilitated in the tricellulin overexpressing group compared to the control group, as reflected by larger tumor volumes (**Figure 7A**), faster tumor growth kinetics (**Figure 7B**), and higher tumor weights (**Figure 7C**).

IHC analysis of xenograft tumors demonstrated dramatically higher levels of tricellulin in Lv-tricellulin group (**Figures 7D, E**). More importantly, elevated tricellulin was accompanied by increased levels of TGFβ1 and phosphorylated SMAD2/3. These findings suggest tricellulin promotes activation of TGFβ1/SMAD2/3 signaling *in vivo*. In addition, upregulated Ki67 in the Lv-tricellulin group indicates it may also facilitate tumor proliferation, which is in consistent with our *in vitro* findings. Moreover, the reduced E-cadherin and increased N-cadherin expression demonstrate that tricellulin promotes CRC metastasis through EMT (**Figures 7D–J**). Collectively, these findings support our *in vitro* data and provide evidence demonstrating that tricellulin facilitates CRC progression by activating the TGFβ/SMAD signaling pathway.

## Discussion

In recent years, there has been a growing interest in the role of tricellulin in tumor progression. However, the mechanisms by which tricellulin may contribute to the progression of CRC specifically remains elusive. In this study, our data show that tricellulin is upregulated significantly in CRC tissues and is associated with poor prognosis. Functionally, we found that tricellulin promotes CRC cell proliferation, migration and EMT. We also provide evidence that that tricellulin promotes the activation of TGFβ1/SMAD2/3 pathway, while TGFβ1 reciprocally regulates tricellulin expression. Consistent with our previous findings (23), nuclear localization of tricellulin in CRC cells indicates a potential role in transcriptional regulation. Meanwhile, in our previous study, we demonstrated that tricellulin expression was significantly associated with tumor metastasis and poor prognosis. In addition, proteomic analysis revealed that tricellulin was involved in EMT and reduction of apoptosis through the NF-κB signaling pathway (23).

Tight junction proteins are critical for maintaining epithelial cell polarity and barrier function, while their dysregulation accelerates the development of cancer (26). As a tricellular tight junction component, tricellulin was considered a structural protein, while its importance in cancer biology has been noticed by researchers (27). Changes in its expression has been shown to promote tumor progression (28). In this study, we evidenced its role in CRC progression.

EMT plays an important role in cancer metastasis. During EMT, epithelial cells lose polarity and cell-cell adhesion and possess a phenotype with higher mobility (23). In alignment with the findings, we observed that tricellulin facilitates EMT in CRC cells. More importantly, this effects was mediated by TGFβ1 signaling, which was supported by previous investigations (29–33).

The TGFβ superfamily, including TGFβs, plays a vital role in cellular activities, such as EMT, migration, and invasion (34). Among them, the TGFβ1/SMAD2/3 signaling pathway plays critical roles in tumor metastasis (35, 36). Of note, TGFβ1 suppresses epithelial growth in normal tissues while promoting tumor progression in advanced cancers (37). The role of TGFβ1 in tumor progression is stage-dependent. Initially, it promotes tumor differentiation and suppresses cell cycle progression and apoptosis. In later stages, it shifts to inducing EMT and thereby promote metastasis (29). TGFβ signaling functions primarily through SMAD pathway (38, 39). Specifically, TGFβ activates SMAD complexes to regulate EMT-related molecules, and inhibits the expression of E-cadherin while promotes the expression of N-cadherin (29). Therefore, we speculate that tricellulin promotes the progression of CRC via TGFβ signaling. Our functional assays confirmed this, demonstrating that tricellulin promotes CRC cell migration and invasiveness. Moreover, these effects were reversible with TGFβ1 activation and SB525334 inhibition.

**Figure 8:**
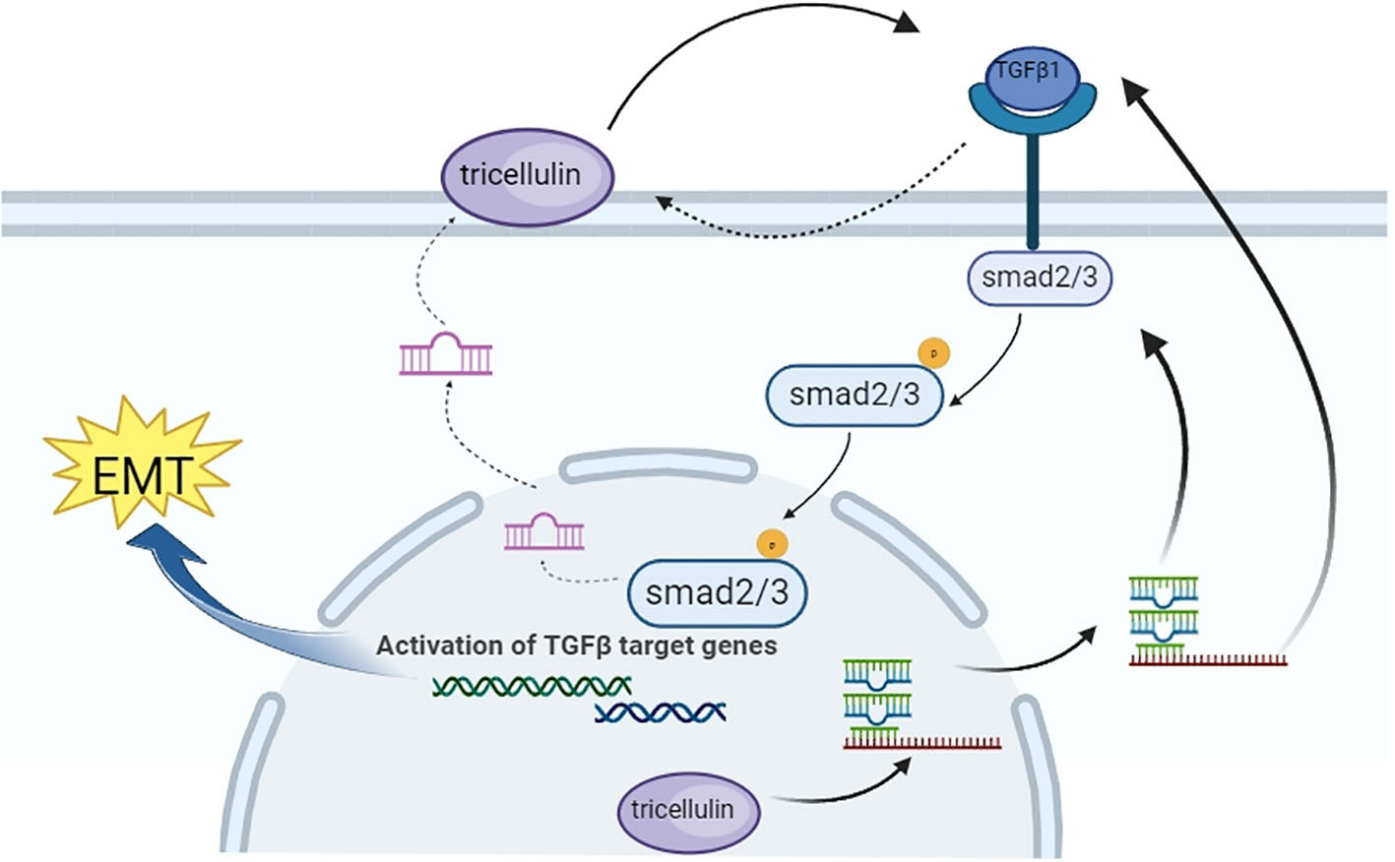
Schematic diagram showing the mechanism by which tricellulin modulates the progression of CRC.

To understand the underlying mechanism, we performed several molecular assays. We found that downregulating tricellulin in HCT116 cells decreased TGFβ1, SMAD2/3, and phosphorylated SMAD2/3 levels, which was rescued by the addition of TGFβ1. Conversely, upregulating tricellulin significantly elevated TGFβ1, SMAD2/3, and SMAD2/3 phosphorylation. Importantly, we found that inhibition of TGFβ/SMAD signaling resulted in a reduction in tricellulin expression, suggesting reciprocal regulation. This is in alignment with previous studies showing that the TGFβ type 1 inhibitor, EW-7197, reduced tricellulin expression in nasal epithelial cells (40), and that TGFβ activation increased tricellulin expression in pancreatic cancer cells (41). These results suggest a positive feedback loop between tricellulin and the TGFβ/SMAD2/3 signaling pathway, which promotes the progression of CRC. Based on our experimental results, we speculate that TGFβ1 signaling affects tricellulin through the p-SMAD2 and p-SMAD3 pathways, as shown in **Figure 8**. TGFβ1 signaling promotes SMAD2 and SMAD3 phosphorylation, leading to their nuclear translocation and activation of tricellulin transcription. In combination with our previous findings (23), the nuclear localization of tricellulin in CRC cells further demonstrates that the regulation of TGFβ1/SMAD2/3 signaling by tricellulin occurs not only at the protein modification level but also at the gt/SMAD pathway. Our research shows that TGFβ signaling is a key upstream regulator in the tricellulin-mediated CRC pathogenesis. In addition, our data show that tricellulin reciprocally regulates TGFβ signaling, forming a positive feedback loop. However, the precise mechanism of this loop requires further investigation.

Apart from the findings, we acknowledge that our study has limitations. First, although we demonstrated that tricellulin activates TGFβ1/SMAD2/3 pathway, direct evidence revealing the molecular mechanism is needed. Second, while TGFβ signaling can activate multiple downstream signaling pathways in addition to SMAD2/3, we focused primarily on the SMAD2/3 axis. A more comprehensive analysis of alternative pathways, such as ERK and JNK pathway, will provide a more complete understanding of the role of tricellulin in CRC progression. Third, our *in vivo* models were limited to subcutaneous xenografts, which lack clinical relevance. Advanced models would provide more convincing results showing the role of tricellulin in CRC metastasis. Despite the limitations, our current findings demonstrate the role of tricellulin in CRC progression and established a foundation for further mechanistic study.

In summary, our study demonstrates that TGFβ1 upregulates tricellulin to promote CRC progression, and that tricellulin activates the TGFβ1/SMAD2/3 signaling pathway, further driving CRC progression. This regulatory mechanism provides a potential therapeutic target in patients with advanced CRC.

## Data Availability

The original contributions presented in the study are included in the article/supplementary material. Further inquiries can be directed to the corresponding authors.
